# Surgical Navigation in Mandibular Reconstruction: Accuracy Evaluation of an Innovative Protocol

**DOI:** 10.3390/jcm11072060

**Published:** 2022-04-06

**Authors:** Davide Sozzi, Andrea Filippi, Gabriele Canzi, Elena De Ponti, Alberto Bozzetti, Giorgio Novelli

**Affiliations:** 1 O.U. Maxillofacial Surgery, Department of Medicine and Surgery, School of Medicine, ASST-Monza, San Gerardo Hospital, University of Milano-Bicocca, Via Pergolesi 33, 20900 Monza, Italy; andreafilippimd@gmail.com (A.F.); alberto.bozzetti@unimib.it (A.B.); nove.gio@gmail.com (G.N.); 2 Post-Graduate School of Maxillofacial Surgery, Department of Medicine and Surgery, University of Milan, Via Festa del Perdono 7, 20122 Milan, Italy; 3 Maxillofacial Surgery Unit, Emergency Department, ASST-GOM Niguarda, Niguarda Hospital, Piazza Ospedale Maggiore 3, 20162 Milan, Italy; gabriele.canzi@ospedaleniguarda.it; 4 Department of Medical Physics, ASST-Monza, San Gerardo Hospital, University of Milano-Bicocca, Via Pergolesi 33, 20900 Monza, Italy; elena.deponti@unimib.it

**Keywords:** mandibular reconstruction, fibula flap, virtual surgical planning, surgical navigation, computer-assisted surgery, oral cancer

## Abstract

**Aim**: the purpose of this work is to present an innovative protocol for virtual planning and surgical navigation in post-oncological mandibular reconstruction through fibula free flap. In order to analyze its applicability, an evaluation of accuracy for the surgical protocol has been performed. **Methods**: 21 patients surgically treated for mandibular neoplasm have been included in the analysis. The Brainlab Vector Vision 3.0^®^ software for surgical navigation has been used for preoperative surgical planning and intra-operative navigation. A post-operative accuracy evaluation has been performed matching the position of mandibular landmarks between pre-operative and post-operative CT scans. **Results**: the maximal discrepancy observed was included between −3.4 mm and +3.2 mm, assuming negative values for under correction and positive values for overcorrection. An average grade of accuracy included between 0.06 ± 0.58 mm and 0.43 ± 0.68 mm has been observed for every mandibular landmark examined, except for mandibular angles that showed a mean discrepancy value included between 1.36 ± 1.73 mm and 1.46 ± 1.02 mm when compared to preoperative measurements. **Conclusion**: a satisfying level of accuracy has been observed in the protocol presented, which appears to be more versatile if compared to closed custom-made systems. The technique described may represent a valid option for selected patients, but it cannot be considered for routine activity because of the complexity of the method, the mobility of the jaw, the necessity of surgical navigator and the long surgical learning curve that is required.

## 1. Introduction

Mandibular reconstruction has been a major challenge for a long time and it is still characterized by considerable complexity, due to the peculiar anatomical morphology and the central role of the jaw in face functions and aesthetics [1,2]. The main field of application for mandibular reconstruction is represented by oncological pathology (benign and malignant), which can involve bone and soft tissue, requiring massive mandibular resections in order to guarantee radicality. Further issues come from complementary radiotherapy, which may induce fibrosis and vascular damage, worsening the functional outcomes [3,4].

Reconstructive surgery underwent a huge development during the last 40 years thanks to the introduction of new surgical techniques, technologies and materials, which converted a highly disfiguring surgery into an effective surgery for both therapeutic and aesthetic purposes [1,5].

The osteocutaneous fibula free flap with titanium implants fixation currently represents one of the best reconstructive options for mandibular defects because of its versatility, the massive portion of bone available and the possibility of being prosthetically rehabilitated [6,7,8,9,10,11,12,13]. A fundamental requirement in order to obtain a good morphological reconstruction is represented by the respect of individual dental occlusion and condyle-glenoid relationship through the accurate shaping of fixation plate that will keep the bone in the correct position.

The evolution of technologies led to the introduction of virtual surgical planning which, through CAD-CAM systems, provides an important aid to the surgeon in both ablative and reconstructive surgical steps [14,15,16,17]. Surgical navigation represents a valid and useful technique for the correct reconstruction of facial middle third, which is represented by non-motile bones; nevertheless, its use in mandibular reconstruction is limited by bone movements that reduce the accuracy of the method [18,19,20,21].

The aim of this study is to evaluate the accuracy of an innovative surgical protocol in mandibular post-oncological reconstruction in order to implement reconstructive outcomes and define the limits of the technique: this work analyzes a single-center experience in computer-assisted planning associated to surgical navigation for one-stage oncological mandibular resection and reconstruction through osteocutaneous fibula free flap.

## 2. Materials and Methods

### 2.1. Sample Selection

This study analyzes 21 patients affected by oral-mandibular neoplasms (both benign and malignant), 9 female and 12 male, with a mean age of 45.9 ± 15.0 years (range 17–65); a mean age of 33.3 years was observed in the group with benign tumor (range 17–65), while a mean age of 53.8 years was observed in patients with malignant diagnosis (range 36–65).

Every patient has been treated through the same protocol of virtual planning and surgical navigation developed by the Unit of Maxillofacial Surgery of San Gerardo Hospital in Monza—Milano-Bicocca University (Italy) between January 2010 and September 2018. Within the sample, 13 patients were affected by malignant neoplasms (11 squamous carcinomas, one synovial sarcoma and one adenoid-cystic carcinoma); eight patients of the whole sample were affected by benign neoplasm (6 ameloblastomas, one odontogenic keratocyst and one giant cell granuloma).

Every patient underwent hemi-mandibulectomy and one-stage reconstruction through free fibula flap. In this case, 14 patients have been treated by resection of ascending and horizontal mandibular branches, with a 2-segments fibular reconstruction; four patients have been treated through the same resection and a double barrel reconstruction; three patients required even partial symphysis resection and a 3-segments fibular reconstruction.

All surgical procedures included in the analysis have been performed by the same team, with the same two senior surgeons cooperating during both resective and reconstructive steps.

### 2.2. Virtual Planning

The first step in surgical planning is represented by the realization of a dental splint, which has the function to stabilize the dental arches and to incorporate the radiopaque marker points for surgical navigation. The splint should be acquired in centric occlusion and it has to be thin enough to allow the correct interfacing of dental cusps and thick enough to maintain integrity during surgical procedure.

**Figure 1:**
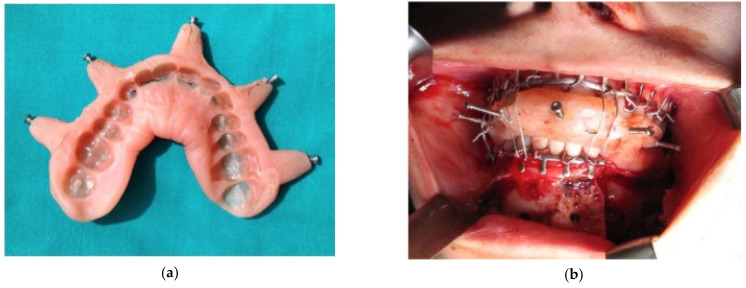

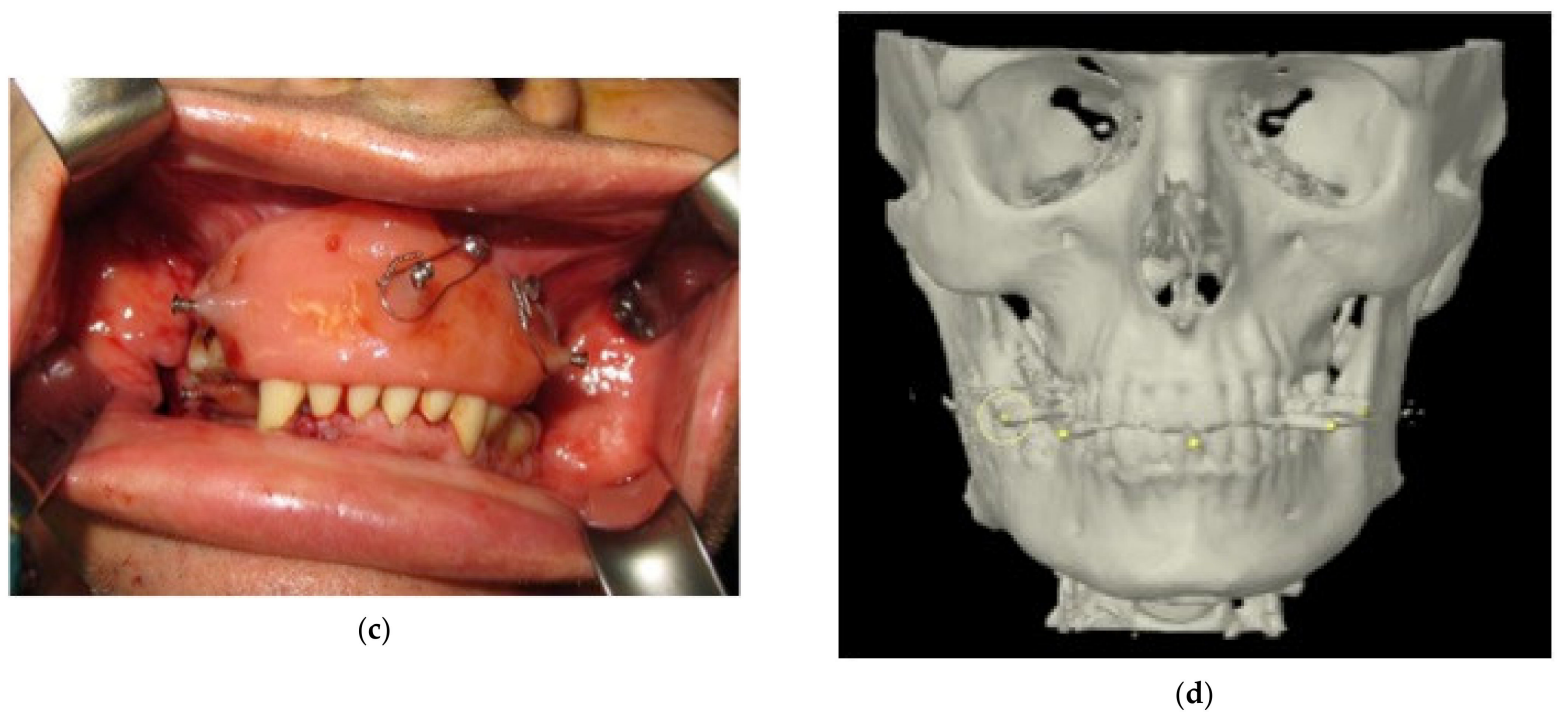
(**a**–**d**): The dental splint is manufactured in order to include the fiducial markerpoints and to maintain a rigid intermaxillary fixation.

The dental splint plays a fundamental role because it contains the fiducial marker points used for surgical navigation and it keeps the mandible in the correct position: the mobility of the mandible during the pre-operative CT scanning and during the intra-operative surgical navigation would make the technique unusable. It is necessary to identify the marker points’ position before surgery and this position must be kept unchanged during every step of the protocol (Figure 1).

Dental splints, created with cold sterilizable acrylic resin, can be modeled on dental arches plaster models (indirect technique) or straight on patient’s dental arches (direct technique). The markers applied on the splint are represented by titanium screws with hexagonal engagement (1.5 mm diameter and 2.0 mm depth). In order to obtain a good spatial reference, it is necessary to place at least five orthogonal marker points in an easy-to-reach position during the entire surgical procedure. The dental splint is then sterilized through gamma-irradiation for the intraoperative use.

The second step of virtual planning is represented by the acquisition of high resolution CT scans of facial bones and lower limbs angio-CT, which have to be elaborated in DICOM (Digital Imaging and Communications in Medicine) format.

Lower limbs angio-CT has a dual purpose: the evaluation of lower limbs vascular anatomy and the structural analysis of the fibular bone for virtual planning.

The facial CT scan must be acquired while the patient is wearing the dental splint and, to ensure maximal accuracy during the virtual planning phase, our protocol provides a laser scan of the dental arches surface, which is lately elaborated and integrated to previously acquired data, reducing scattering artifacts that can be encountered in CT images.

**Figure 2:**
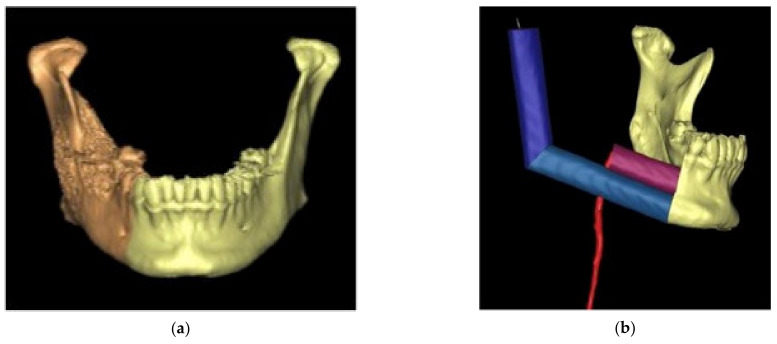
(**a**,**b**): Virtual surgical planning on CT images.

Images are then imported in the planning software and converted into STL (Standard Triangulation Language) format, which allows to elaborate an accurate 3D model that can be manipulated in order to simulate the surgical resection. After that, the virtual 3D fibula is segmented and modeled through the same software in order to replenish the mandibular continuity (Figure 2).

### 2.3. 3D Model Printing and Plate Shaping

**Figure 3:**
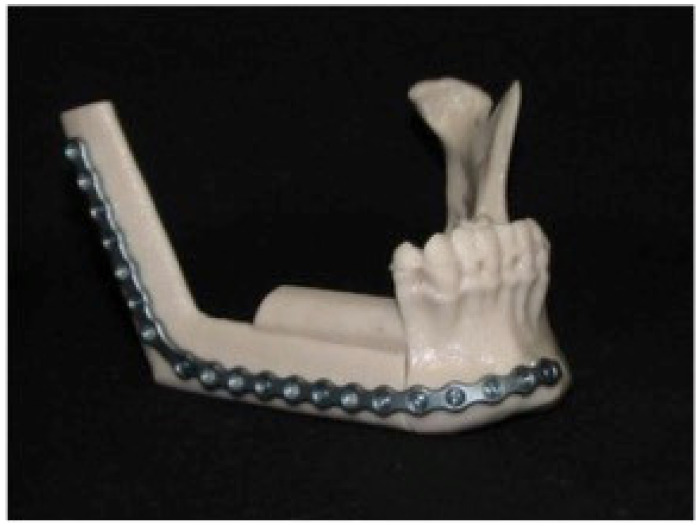
The titanium plate is shaped on 3D printed model.

The final model of the “new” mandible is printed through a 3D-printer using thermoplastic powders and it is used as template for reconstructive titanium plate shaping (Figure 3).

### 2.4. Pre-Surgical Navigation: 3D Model Registration

CT images and virtual planning data are imported to the Brainlab Vector Vision 3.0^®^ software for surgical navigation and every marker point position is then identified on the CT images to make triangulation possible.

The Dynamic Reference Frame (DRF) system is attached to the 3D mandible model: it is represented by a tripod on which are fixed some luminous reflectors, that are detected by the infrared camera of the surgical navigator and permit the identification of the spatial position of the 3D model. The DRF system can guarantee high accuracy of surgical navigation even in a mobile body region, such as the patient’s head.

**Figure 4:**
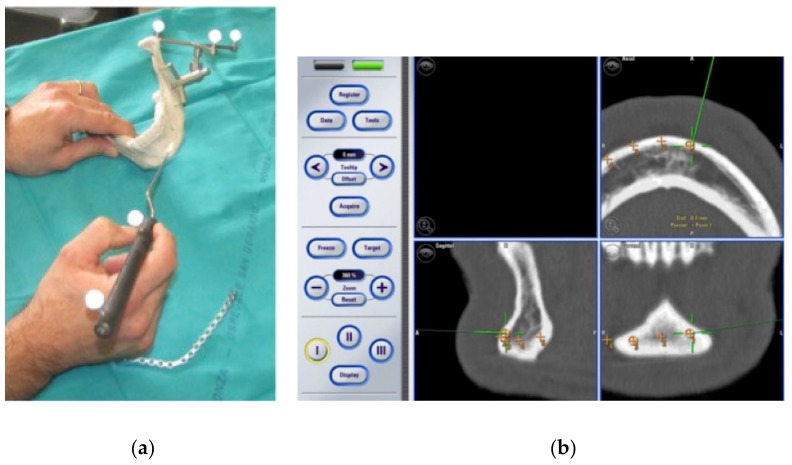
(**a**,**b**): Through preoperative surgical navigation of the 3D model, the position of the screw holes is recorded.

The position of the screw holes previously realized on the 3D model is then recorded, as well as the lines of resection that are expected to be followed in the ablative surgical step (Figure 4).

### 2.5. Intra-Operative Surgical Navigation

The intra-operative surgical navigation begins with the positioning of the DRF system tripod on patient’s skull and the subsequent placing of the dental splint on patient’s dental arches; marker points position on dental splint is then recorded and matched with CT scans previously loaded. 

In this phase every surgical instrument should be recorded using a dedicated calibration tool which recognizes type, dimensions and characteristics of the different surgical instruments.

**Figure 5:**
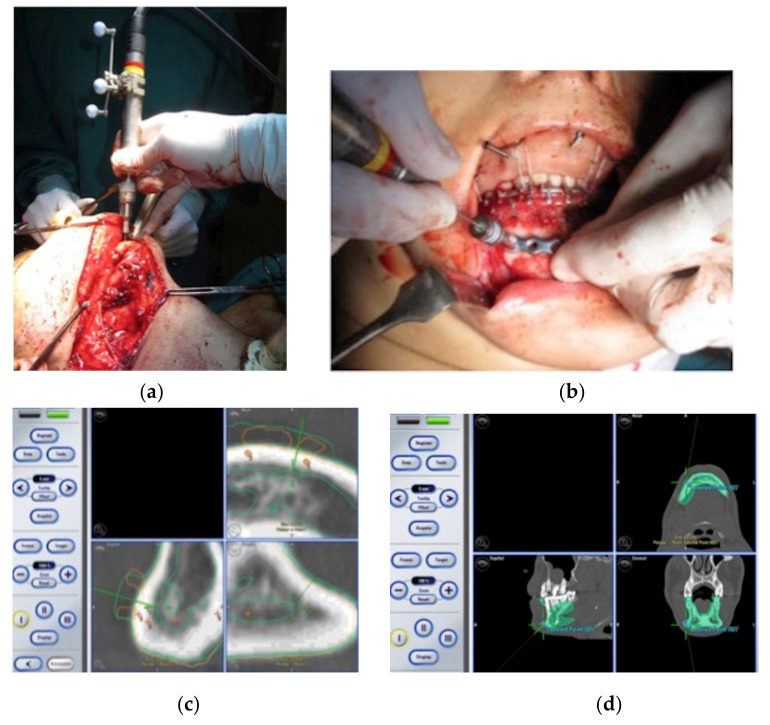
(**a**–**d**): The fibula flap is inset and fixed through a recorded drill and surgical navigation.

The mandibular bone is exposed and, before performing the resection, the screw holes for reconstruction plate are realized with a recorded drill, whose bit position can be identified live on CT images in order to place the screws in the exact programmed location (Figure 5). The mandibular resection is then performed respecting the programmed margins, following the oncological safety principle.

Once the pathological bone segment has been removed, the reconstruction plate should be placed, matching the position of the holes made with the positions previously programmed. Simultaneously, a second surgical team performs the fibula flap harvesting [6]. When flap preparation is complete, the planned osteotomies are performed and the fibular bone is shaped with the aid of the 3D plaster model.

The modeled bone is then inset onto the receiving site, precisely adapting it to the shape of the titanium plate, which works as rigid template for the final modeling of the reconstructive bone. A final check of the bone and plate position is made to guarantee a good matching between the virtual planning and the new mandible. Lastly, the flap is revascularized performing microsurgical anastomoses with the most fitting cervical-facial vessels.

### 2.6. Post-Operative Evaluation

**Figure 6:**
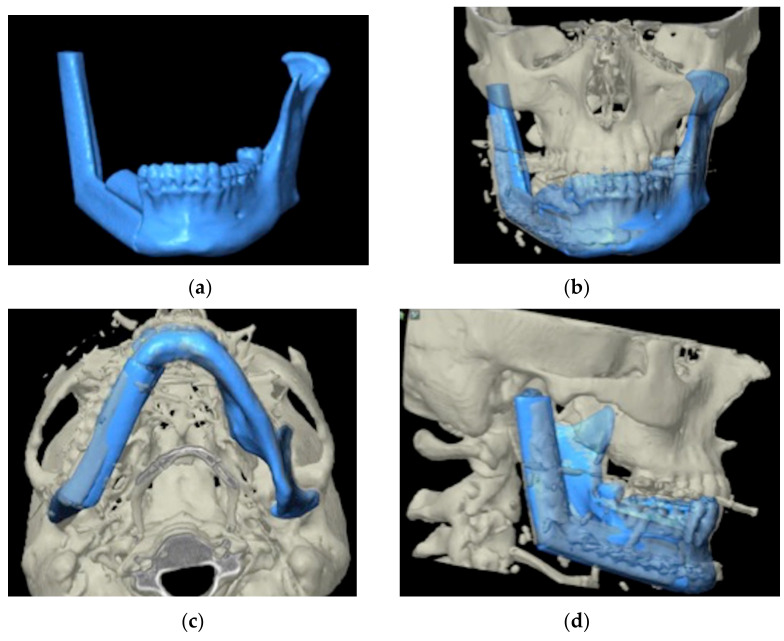
(**a**–**d**): Preoperative images of surgical planning and postoperative CT images are overlapped and positional discrepancies are measured.

The post-operative evaluation is based on the overlap of post-operative CT images with the pre-operative virtual planning images through the Plasticad 3DIEMME 2015^®^ software, realizing tridimensional virtual STL models and analyzing the matching between the overlapped models; the difference in millimeters for specific structures is then calculated (Figure 6).

In this work, the post-operative CT scan has been acquired within 15 days from surgery and the correspondence analysis focused on the residual mandible, assuming a value equal to 0 in case of perfect overlap and match, a negative value in case of undercorrection and a positive value in case of overcorrection. The virtual model overlap should be analyzed in all spatial planes.

The anatomical structures that have been used for the overlap analysis are: left condyle (sagittal and coronal projection), right condyle (sagittal and coronal projection), mandibular midline (sagittal and coronal projection), left mandibular angle (sagittal and coronal projection) and right mandibular angle (sagittal and coronal projection).

### 2.7. Statistical Analysis

The surgical data and image measures were retrospectively collected and analyzed. The parameters of evaluation have been obtained calculating the difference in millimeters between post-operative and pre-operative position of reference points. The statistical analysis has been performed through the Stata 9.0^®^ software (Stata Corporation College Station, TX, USA). Mean and median of every parameter have been calculated.

The distribution of the values has been verified through indexes of symmetry (*Skewness Index*), shape (*Kurtosi Index*) and global normality.

All variables measuring accuracy were characterized by Gaussian distribution, except for the parameter “*right mandibular angle in coronal projection*”, which resulted asymmetrical. This fact confirmed that mean was the correct indicator to represent all the observations, except for “*right mandibular angle in coronal projection*”, for which median and Wilcoxon sum rank test was used.

The normal data distribution enabled to use the parametric Student’s *t*-test for the comparison of the means of all the other parameters evaluated.

The second step of data analysis has been led evaluating the matching between the treated side for each patient and the offset value showing the least accuracy, examining differences related to the side of the surgical site.

Lastly, it has been checked whether the average of the individual measured parameters significantly deviated from zero, which represents the absolute perfection of the surgical outcome, separately considering patients operated on left-hand and right-hand side. This analysis was performed applying the Student’s *t*-test.

The level of significance used for the study of the associations described was assumed for a *p* value < 0.05.

## 3. Results

**Figure 7:**
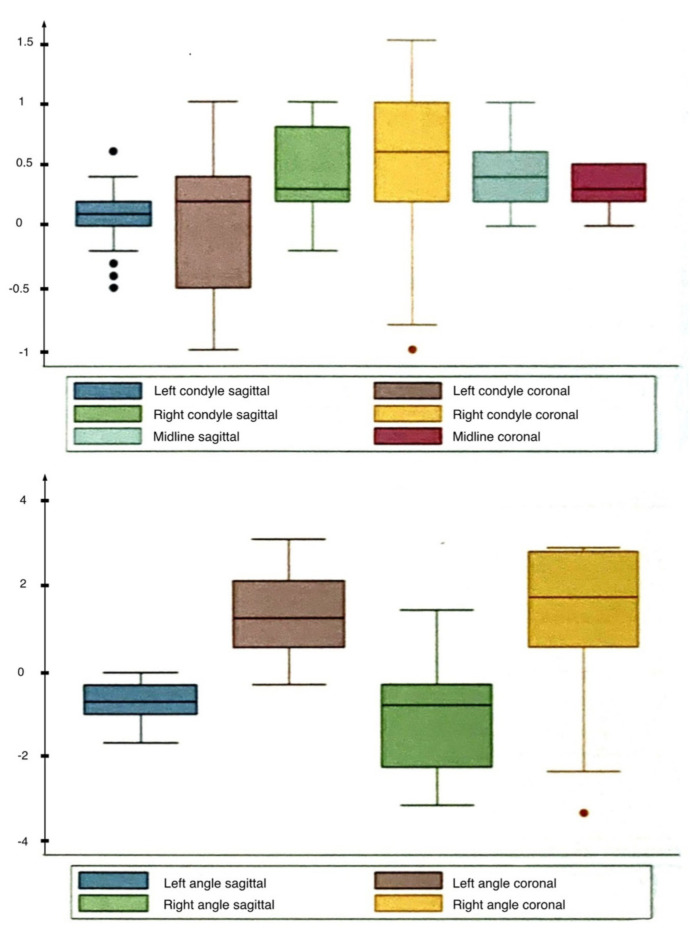
Landmarks box plot: the box represents the interquartile range between Q1 and Q3, the continuous line inside the box represents the median, the continuous line outside the box shows the global range of parameters distribution and the dots represent outliers.

The quantitative features of accuracy parameter have been displayed through a box plot in order to evaluate the distribution of the parameters (Figure 7). The box plot analysis showed a Gaussian distribution of the results, except for “right mandibular angle in coronal projection”, which revealed a highly asymmetrical distribution. An overcorrective tendency has been observed for the parameters “right condyle (sagittal and coronal projection)”, “midline (sagittal and coronal projection)”, “left angle in coronal projection” and “right angle in coronal projection”; an undercorrective tendency has been observed instead for “left angle in sagittal projection” and “right angle in sagittal projection”.

A global error between −3.4 mm and +3.2 mm has been observed in the entire examination, with a higher grade of discrepancy documented for the mandibular angles position (Table 1).

In order to detect significant differences of accuracy between patients treated on right and left-hand sides, the mean values of every marker point have been compared through Student’s *t*-test (Table 2).

Accuracy parameters have been finally compared with “zero”, which is considered as the condition of perfect matching between preoperative and postoperative marker points’ position. Once again, the Student’s *t*-test has been used for this analysis (Table 3 and Table 4).

No perfect matching has been observed for both treated sides (examined separately) and the highest discrepancy has been detected for mandibular angles in coronal projection: 2.29 ± 1.19 mm for left-hand side and 1.65 ± 2.04 mm for right-hand side.

## 4. Discussion

Free flaps represent the gold standard for post-oncological mandibular reconstruction and the fibula flap, with its reliable vascular pedicle and its reduced donor site morbidity, provides an adequate quantity of bicortical bone that is suitable for dental rehabilitation [6,10,11,12,13,14,22,23,24,25,26,27]. While “*free-hand*” techniques are characterized by operator-dependent outcomes and by less accuracy in condylar repositioning and individual occlusion restoration [14,22], the introduction of Computer-Assisted Surgery (CAS) techniques led to more accurate reconstructions and to a shorter length of surgery [14,17,28,29].

A further evolution of techniques is represented by the integration of Computer-Assisted Design (CAD) and Computer-Assisted Manufacturing (CAM): the CAD-CAM technique has led to the introduction of *Patient-Specific Mandible Reconstruction Plates* (*PSMPs*) and surgical cutting guides [15,30,31]. Although this process provides a high grade of accuracy in bone reconstruction and it is widely used [32], it should be considered that such a rigid system may lead to various issues when dealing with malignant tumors that may require to modify the resection width.

Maxillofacial surgical navigation has been firstly introduced in 1994 by Hassfeld for the treatment of skull base tumors [33] and then it has been successfully applied to orbit, temporal bone, zygomatic bone, and maxillary bone [20,34,35,36,37,38,39,40]; all these sites are characterized by immobility. The surgical navigation of the mandible was considered difficult and impractical because of the jaw mobility: in 2011 Bell et al. elaborated a protocol of virtual planning and surgical navigation for maxillary and mandibular bone reconstruction coming to the conclusion that mandibular surgical navigation is not superior to the method with CAD-CAM models, but rather it introduces more complexity and increased surgical time [41]. Considering the current limits of CAD-CAM techniques, related to the rigidity of the system and the need of involving qualified engineers in the preoperative steps, we decided to exploit our experience with surgical navigation in treatment of upper and middle third of the skull in order to elaborate a new protocol of integration between virtual surgery simulation (VSS) and surgical navigation, evaluating its accuracy [30,31,32,38,39,40]. Every step of our protocol has been performed by physicians, with no need of further professional figures.

The main problem of surgical navigation of the jaw is represented by the mobility of the mandibular bone and the consequent difficulty in placing and identifying the fiducial marker points [41,42,43,44].

Wu et al. (2016), Zhang el al. (2016) and Shan et al. (2016) described the use of a custom-made dental splint to improve stability and reproducibility of inter-maxillary fixation during the different phases. Marker points were placed, in form of titanium screws, on maxillary bone and were not integrated to the dental splint [18,32,42].

Within this background, we elaborated a protocol that provides the use of a dental splint on which radiopaque fiducial marker points are built-in. The splint and mandible positions must necessarily be the same during the phase of CT images acquisition and the surgical phase, in order to ensure a perfect matching between patient and radiological data. In order to obtain spatial stability, the mandible and the splint are maintained in the same position during the whole surgical procedure through the use of inter-maxillary rigid fixation.

Basic importance for the realization of the procedure is represented by the precision of the dental splint and the 3D model, which is made through a *laser sintering* technique and has to perfectly match with the dental splint. We elaborated an intra-oral laser scan system for both the dental arches that produces DICOM format images, which are superimposed to high resolution CT images of the skull bone; this system ensures high accuracy for the virtual and 3D sintered models, significantly reducing the scattering artifacts.

The use of a dental splint with built-in markers points allows the maintenance of a stable and reproducible maxillary-mandibular centric occlusion and to acquire easy access to the marker points during both the planning and surgical phases, with high accuracy and a systematic method-related error lower than 1 mm [20].

We consider that splint-linked marker points are a valuable tool for surgical navigation of the jaw because of the low invasiveness and the high accessibility; these marker points also allow the surgeon to obtain a stable inter-maxillary fixation even after the oncological resection.

Further surgical navigation methods have been previously described [19,32,42,43,44]: some Authors suggested the use of an inter-maxillary fixation without dental splint, with lower accuracy and reproducibility of the procedure. Casap et al. in 2008 and Abbate et al. in 2017 introduced the positioning of the DRF system directly on the mandible, opposite to the lesion side, obtaining a good matching between the post-operative outcome and the virtual planning; although this method may represent a valid alternative to the use of inter-maxillary fixation, it is characterized by some important disadvantages, such as the long time required for the procedure, the possibility of screws loosening on the DRF system and the size of the space occupied by the device in the operative field [19,44].

In our experience, the application of the DRF system on the patient’s skull appears to be the most adequate solution in combination with the use of a dental splint and intra-operative inter-maxillary fixation.

The main aim of this work was to assess the accuracy of an innovative protocol analyzing the position of reference point on CT pre-operative and post-operative images.

No flap failure has been observed in the sample studied. The post-operative clinical evaluation showed a satisfactory functional outcome, with restoration of the jaw symmetry and dental-skeletal occlusion; the mutual position of condyle in the glenoid cavity led to an adequate mandible mobility in all patients.

The accuracy assessment has been performed, as already proposed by Roser et al., by overlapping post-operative CT images and virtual planning data, and then comparing the position of reference point [22]. The reference points that have been selected for the overlapping analysis are: left condyle, right condyle, midline, left mandibular angle and right mandibular angle (in sagittal and coronal projection). These points have been identified because of the aesthetic and functional relevance in mandibular reconstruction: the condyle position is essential to prevent malocclusion, mobility defects, chronic pain and temporomandibular ankylosis; the midline position is important for symmetry and occlusion; mandibular angles are highly relevant for facial aesthetics and masticatory load distribution.

The surgical navigation system showed a systematic error lower than 1 mm after the correct registration of marker points, coinciding with literature data [18,45]. In the analysis of postoperative measures negative values have been associated to undercorrection, while positive values represent overcorrection.

The means of the accuracy assessment parameters showed a lower precision in the angles repositioning, with a mean between 1.36 ± 1.73 mm and 1.46 ± 1.02 mm (Table 1). We consider this outcome as an acceptable one because this difference in the mandibular district has no adverse clinical implications, with a good aesthetic and functional result.

We observed a higher accuracy for the rest of the reference points analyzed, averaging between 0.06 ± 0.58 mm and 0.43 ± 0.68 mm (Table 1).

Our results agree with literature data, which show a mean overlapping precision between 1 and 3 mm [42]. Wu et al. analyzed the accuracy of mandibular angles repositioning observing a mean result of 1.92 ± 0.97 mm [18]. Zhang et al. proposed a protocol for surgical navigation in mandibular reconstruction through the use of iliac crest free flap and reported a mean difference in condyle positioning of 1.45 ± 0.50 mm [32].

All studied parameters did not show correlation with the treated side of the jaw, confirming the absence of significant systematic errors of the procedure deriving from the side of the surgical site.

The comparison between mean and median values of the studied parameters divided by side of surgical site showed a condyle overcorrection trend in sagittal projection for patients treated on left-hand side (left condyle *p* value = 0.030 and right condyle *p* value = 0.022), an angle undercorrection trend in sagittal projection (left angle *p* value < 0.0001 and right angle *p* value = 0.078) and an angle overcorrection trend in coronal projection (left angle *p* value = 0.002 and right angle *p* value = 0.023) (Table 2). This trend may be induced by the presence of the reconstructive titanium plate, which produces a slight thickness and induces a higher overlapping difference value in coronal projection. The sagittal undercorrection may be explained instead by the morphology of the reconstructive bone, which turns out to be more edgy and less uniform.

The comparison between the means of single parameters and the “*zero*” value, intended as the perfect overlap and then the theoretical perfect reconstruction, showed that the accuracy of our protocol, although not achieving perfection, turns out to be highly satisfying.

The difference in overlap for midline in sagittal projection may be explained again by the presence of the reconstructive plate, considering the value of the difference that is equal to 0.43 ± 0.41 mm (*p* value = 0.033) for left-hand side and 0.46 ± 0.25 mm (*p* value < 0.0001) for right-hand side (Table 3 and Table 4).

This study has been performed on a 21 patients sample, which appears to be a relevant and conspicuous number when compared to international scientific literature [18,19]; nevertheless, the absolute number of the sample examined is exiguous and does not permit the obtainment of a satisfying statistical significance. Further studies on larger samples should be performed in the future with the aim of confirming and validating the results.

Although virtual planning and surgical navigation may present several advantages for reconstruction accuracy and length of surgery, it is mandatory to highlight that pre-operative surgical planning and 3D model production require a massive use of time and resources.

A further advantage of surgical navigation, which emerged from this study and was also asserted by Wu et al., concerns the versatility of the technique, that can be adapted to the margins of resection required by clinical condition, leading to an accurate reconstruction even in case of resection modifications [18]. This element shows important relevance when dealing with cancer patients, since the necessity of widened resections may occur in order to achieve a correct treatment from an oncological point of view.

One of the main limits of this protocol is represented by the relevant time necessary for pre-operative virtual planning and registration, which is highly augmented if compared to free-hand techniques. Another simple but basic limit of this technique is represented by the need of the surgical navigator, which nowadays is not used by every hospital facility.

An important practical limit of the method is represented by very bulky tumor mass and edentulous patients: a correct placing of the dental splint and a stable inter-maxillary fixation cannot be obtained in these patients.

Surgical navigation requires a long and complex learning curve, implying a reduced diffusion of the method. Moreover, “Computer-Assisted Surgery” techniques, virtual planning and surgical navigation represent an important aid for the surgeon but do not replace the personal experience, which retains its essentiality.

## 5. Conclusions

The protocol of virtual planning and surgical navigation elaborated by our team represents an innovation for mandibular reconstruction and showed high standards of accuracy.

Virtual planning simulates the surgical phase in order to obtain highly predictable and reproducible outcomes; it constitutes the basis of the surgical navigation. The real time guide offered by this technique reduces the margin of error compared to free-hand techniques, length of surgery is reduced and, furthermore, surgical navigation allows the surgeon to modify ongoing the resection and reconstruction dimensions.

The proposed virtual planning and surgical navigation protocol showed high accuracy and good applicability, but it should be emphasized that it requires massive pre-surgical time of application and a long learning curve for the surgeon.

Even if it should be reserved for selected cases, this protocol may represent a valid alternative surgical option for mandibular reconstruction, leading to satisfactory outcomes with reduction of the length of surgery.

## Figures and Tables

**Table 1:** Mean and median values of difference (in mm) between pre-operative and post-operative position of every mandibular marker point, measured on TC images.

MARKERPOINTS	MEAN ± SD (Range)	MEDIAN (95%CI)
Left condyle sagittal (mm)	0.07 ± 0.28 (−0.5; 0.6)	0.1 (0.0:0.2)
Left condyle coronal (mm)	0.06 ± 0.58 (−1.0; 1.0)	0.2 (−0.5; 0.4)
Right condyle sagittal (mm)	0.4 ± 0.38 (−0.2; 1.0)	0.3 ± (0.2; 0.7)
Right condyle coronal (mm)	0.43 ± 0.68 (−1.0; 1.5)	0.6 (0.2; 1.0)
Midline sagittal (mm)	0.45 ± 0.30 (0.0; 1.0)	0.4 (0.2; 0.6)
Midline coronal (mm)	0.29 ± 0.17 (0.0; 0.5)	0.3 (0.2; 0.5)
Left angle sagittal (mm)	−0.7 ± 0.48 (−1.7; 0.0)	−0.7 (−1.0; −0.3)
Left angle coronal (mm)	1.46 ± 1.02 (−0.3; 3.2)	1.3 (0.6; 2.1)
Right angle sagittal (mm)	−1.07 ± 1.42 (−3.2; 1.5)	−0.8 (−2.2; −0.3)
Right angle coronal (mm)	1.36 ± 1.73 (−3.4; 3.0)	1.8 (0.7; 2.8)

**Table 2:** Comparison of mandibular marker points through Student’s *t*-test for patients treated on right-hand and left-hand side.

MARKERPOINTS	LEFT (*n* = 7) Mean ± SD (Range) *Median* (95% CI)	RIGHT (*n* = 14) Mean ± SD (Range) *Median* (95% CI)	*p* Value Student’s *t*-Test * Wilcoxon Sum Rank Test
Left condyle sagittal (mm)	0.23 ± 0.21 *0.2 (−0.1; 0.4)*	−0.01 ± 0.29 *0.0 (−0.3; 0.2)*	0.030
Left condyle coronal (mm)	0.01 ± 0.51 *0.2 (−0.3; 0.4)*	0.08 ± 0.63 *0.2 (−0.5; 0.7)*	0.409
Right condyle sagittal (mm)	0.63 ± 0.28 *0.6 (0.3; 0.9)*	0.28 ± 0.38 *0.3 (−0.1; 0.5)*	0.022
Right condyle coronal (mm)	0.53 ± 0.68 *0.8 (0.2; 1.0)*	0.39 ± 0.70 *0.5 (−0.3; 1.0)*	0.332
Midline sagittal (mm)	0.43 ± 0.41 *0.3 (0.1; 1.0)*	0.46 ± 0.25 *0.4 (0.2; 0.6)*	0.404
Midline coronal (mm)	0.30 ± 0.18 *0.3 (0.2; 0.5)*	0.29 ± 0.17 *0.3 (0.1; 0.5)*	0.432
Left angle sagittal (mm)	−1.21 ± 0.31 *−1.2 (−1.5; −0.9)*	−0.44 ± 0.31 *−0.3 (−0.7; −0.2)*	<0.0001
Left angle coronal (mm)	2.29 ± 1.19 *2.6 (2.2; 3.0)*	1.04 ± 0.62 *1.1 (0.5; 1.5)*	0.002
Right angle sagittal (mm)	−0.44 ± 0.35 *−0.5 (−0.8; −0.3)*	−1.39 ± 1.65 *−1.8 (−2.9; −0.1)*	0.078
Right angle coronal (mm)	0.77 ± 0.62 *0.6 (0.4; 1.2)*	1.65 ± 2.04 *2.4 (1.5; 2.9)*	0.023 *

Every value of the column was calculated through Student’s *t*-test, except the last value (Right angle coronal) which was calculated through Wilcoxon Sum Rank Test (that’s the reason of *).

**Table 3:** Comparison between mean values of marker points measure and “zero” for patients treated on left-hand side. *p* value < 0.5 has been considered significant.

MARKERPOINTS	LEFT (*n* = 7) Mean ± SD	*p* Value Student’s *t*-Test
Left condyle sagittal (mm)	0.23 ± 0.21	0.026
Left condyle coronal (mm)	0.01 ± 0.51	0.943
Right condyle sagittal (mm)	0.63 ± 0.28	0.001
Right condyle coronal (mm)	0.53 ± 0.68	0.086
Midline sagittal (mm)	0.43 ± 0.41	0.033
Midline coronal (mm)	0.30 ± 0.18	0.005
Left angle sagittal (mm)	−1.21 ± 0.31	<0.0001
Left angle coronal (mm)	2.29 ± 1.19	0.002
Right angle sagittal (mm)	−0.44 ± 0.35	0.016
Right angle coronal (mm)	0.77 ± 0.62	0.016

**Table 4:** Comparison between mean values of marker points measure and zero for patients treated on right-hand side. *p* value < 0.5 has been considered significant.

MARKERPOINTS	RIGHT (*n* = 14) Mean ± SD	*p* Value Student’s *t*-Test
Left condyle sagittal (mm)	−0.01 ± 0.29	0.854
Left condyle coronal (mm)	0.08 ± 0.63	0.648
Right condyle sagittal (mm)	0.28 ± 0.38	0.016
Right condyle coronal (mm)	0.39 ± 0.70	0.061
Midline sagittal (mm)	0.46 ± 0.25	<0.0001
Midline coronal (mm)	0.29 ± 0.17	<0.0001
Left angle sagittal (mm)	−0.44 ± 0.31	<0.0001
Left angle coronal (mm)	1.04 ± 0.62	<0.0001
Right angle sagittal (mm)	−1.39 ± 1.65	0.008
Right angle coronal (mm)	1.65 ± 2.04	0.010

## Data Availability

The data that support the findings of this study are available on request from the corresponding author. The data are not publicly available due to ethical and privacy restriction.

## References

[1] Bak M., Jacobson A.S., Buchbinder D., Urken M.L. (2010). Contemporary reconstruction of the mandible. Oral Oncol..

[2] Ferreira J.J., Zagalo C.M., Oliveira M.L., Correia A.M., Reis A.R. (2015). Mandible reconstruction: History, state of the art and persistent problems. Prosthet. Orthot. Int..

[3] Schliephake H., Hausamen J.E., Ward Booth P., Schendel S.A., Hausamen J.E. (2007). An overview of the principles of reconstructive surgery. Maxillofacial Surgery.

[4] Wilik R.M., Miloro M. (2004). Bony reconstruction of the jaws. Peterson’s Principles of Oral an Maxillofacial Surgery.

[5] Goh B.T., Lee S., Tideman H., Stoelinga P.J. (2008). Mandibular reconstruction in adults: A review. Int. J. Oral Maxillofac. Surg..

[6] Hidalgo D.A. (1989). Fibula free flap: A new method of mandible reconstruction. Plast. Reconstr. Surg..

[7] Hidalgo D.A., Pusic A.L. (2002). Free-flap mandibular reconstruction: A 10-year follow-up study. Plast. Reconstr. Surg..

[8] Yim K.K., Wei F.C. (1994). Fibula osteoseptocutaneous flap for mandible reconstruction. Microsurgery.

[9] Brown J.S., Lowe D., Kanatas A., Schache A. (2017). Mandibular reconstruction with vascularised bone flaps: A systematic review over 25 years. Br. J. Oral Maxillofac. Surg..

[10] Lodders J.N., Leusink F.K.J., Ridwan-Pramana A., Winters H.A.H., Karagozoglu K.H., Dekker H., Forouzanfar T., Schulten E.A.J.M. (2021). Long-term outcomes of implant-based dental rehabilitation in head and neck cancer patients after reconstruction with the free vascularized fibula flap. J. Craniomaxillofac. Surg..

[11] Sozzi D., Novelli G., Silva R., Connelly S.T., Tartaglia G.M. (2017). Implant rehabilitation in fibula-free flap reconstruction: A retrospective study of cases at 1–18 years following surgery. J. Craniomaxillofac. Surg..

[12] Zavattero E., Ramieri G., Agrò G., Fasolis M., Garzino-Demo P., Borbon C. (2021). Implant Dental Rehabilitation of Fibula-Free Flap Reconstructed Jaws. J. Craniofac. Surg..

[13] Kumar V.V., Jacob P.C., Ebenezer S., Kuriakose M.A., Kekatpure V., Baliarsing A.S., Al-Nawas B., Wagner W. (2016). Implant supported dental rehabilitation following segmental mandibular reconstruction—Quality of life outcomes of a prospective randomized trial. J. Craniomaxillofac. Surg..

[14] Zhang L., Liu Z., Li B., Yu H., Shen S.G., Wang X. (2016). Evaluation of computer-assisted mandibular reconstruction with vascularized fibular flap compared to conventional surgery. Oral Surg. Oral Med. Oral Pathol. Oral Radiol..

[15] Wilde F., Hanken H., Probst F., Schramm A., Heiland M., Cornelius C.P. (2015). Multicenter study on the use of patient-specific CAD/CAM reconstruction plates for mandibular reconstruction. Int. J. Comput. Assist. Radiol. Surg..

[16] Mascha F., Winter K., Pietzka S., Heufelder M., Schramm A., Wilde F. (2017). Accuracy of computer-assisted mandibular reconstructions using patient-specific implants in combination with CAD/CAM fabricated transfer keys. J. Craniomaxillofac. Surg..

[17] Succo G., Berrone M., Battiston B., Tos P., Goia F., Appendino P., Crosetti E. (2015). Step-by-step surgical technique for mandibular reconstruction with fibular free flap: Application of digital technology in virtual surgical planning. Eur. Arch. Otorhinolaryngol..

[18] Wu J., Sun J., Shen S.G., Xu B., Li J., Zhang S. (2016). Computer-assisted navigation: Its role in intraoperatively accurate mandibular reconstruction. Oral Surg. Oral Med. Oral Pathol. Oral Radiol..

[19] Abbate V., Orabona G.D.A., Solari D., Bonavolontà P., Iaconetta G., Califano L. (2017). Mandibular Surgical Navigation: An Innovative Guiding Method. J. Craniofac. Surg..

[20] Hohlweg-Majert B., Schön R., Schmelzeisen R., Gellrich N.C., Schramm A. (2005). Navigational maxillofacial surgery using virtual models. World J. Surg..

[21] Yu H., Shen S.G., Wang X., Zhang L., Zhang S. (2013). The indication and application of computer-assisted navigation in oral and maxillofacial surgery-Shanghai’s experience based on 104 cases. J. Craniomaxillofac. Surg..

[22] Roser S.M., Ramachandra S., Blair H., Grist W., Carlson G.W., Christensen A.M., Weimer K.A., Steed M.B. (2010). The accuracy of virtual surgical planning in free fibula mandibular reconstruction: Comparison of planned and final results. J. Oral Maxillofac. Surg..

[23] Wang L.Y., Du H.M., Zhang G., Tang W., Liu L., Jing W., Long J. (2011). The application of digital surgical diagnosis and treatment technology: A promising strategy for surgical reconstruction of craniomaxillofacial defect and deformity. Med. Hypotheses.

[24] Sink J., Hamlar D., Kademani D., Khariwala S.S. (2012). Computer-aided stereolithography for presurgical planning in fibula free tissue reconstruction of the mandible. J. Reconstr. Microsurg..

[25] He Y., Zhu H.G., Zhang Z.Y., He J., Sader R. (2009). Three-dimensional model simulation and reconstruction of composite total maxillectomy defects with fibula osteomyocutaneous flap flow-through from radial forearm flap. Oral Surg. Oral Med. Oral Pathol. Oral Radiol. Endod..

[26] Lyons A.J., James R., Collyer J. (2005). Free vascularised iliac crest graft: An audit of 26 consecutive cases. Br. J. Oral Maxillofac. Surg..

[27] Muñoz Guerra M.F., Gías L.N., Rodríguez Campo F.J., Díaz González F.J. (2001). Vascularized free fibular flap for mandibular reconstruction: A report of 26 cases. J. Oral Maxillofac. Surg..

[28] Hendra F.N., Van Cann E.M., Helder M.N., Ruslin M., de Visscher J.G., Forouzanfar T., de Vet H.C.W. (2020). Global incidence and profile of ameloblastoma: A systematic review and meta-analysis. Oral Dis..

[29] Moratin J., Horn D., Metzger K., Ristow O., Flechtenmacher C., Engel M., Hoffmann J., Freier K., Freudlsperger C. (2020). Squamous cell carcinoma of the mandible—Patterns of metastasis and disease recurrence in dependence of localization and therapy. J. Craniomaxillofac. Surg..

[30] Wilde F., Cornelius C.P., Schramm A. (2014). Computer-Assisted Mandibular Reconstruction using a Patient-Specific Reconstruction Plate Fabricated with Computer-Aided Design and Manufacturing Techniques. Craniomaxillofac. Trauma Reconstr..

[31] Wilde F., Winter K., Kletsch K., Lorenz K., Schramm A. (2015). Mandible reconstruction using patient-specific pre-bent reconstruction plates: Comparison of standard and transfer key methods. Int. J. Comput. Assist. Radiol. Surg..

[32] Zhang W.B., Yu Y., Wang Y., Mao C., Liu X.J., Guo C.B., Yu G.Y., Peng X. (2016). Improving the accuracy of mandibular reconstruction with vascularized iliac crest flap: Role of computer-assisted techniques. J. Craniomaxillofac. Surg..

[33] Hassfeld S., Mühling J., Zöller J. (1995). Intraoperative navigation in oral and maxillofacial surgery. Int. J. Oral Maxillofac. Surg..

[34] Hassfeld S., Mühling J. (2001). Computer assisted oral and maxillofacial surgery--a review and an assessment of technology. Int. J. Oral Maxillofac. Surg..

[35] Austin R.E., Antonyshyn O.M. (2012). Current applications of 3-d intraoperative navigation in craniomaxillofacial surgery: A retrospective clinical review. Ann. Plast. Surg..

[36] Bell R.B. (2010). Computer planning and intraoperative navigation in cranio-maxillofacial surgery. Oral Maxillofac. Surg. Clin. N. Am..

[37] Yu H., Shen G., Wang X., Zhang S. (2010). Navigation-guided reduction and orbital floor reconstruction in the treatment of zygomatic-orbital-maxillary complex fractures. J. Oral Maxillofac. Surg..

[38] Novelli G., Tonellini G., Mazzoleni F., Sozzi D., Bozzetti A. (2012). Surgical navigation recording systems in orbitozygomatic traumatology. J. Craniofac. Surg..

[39] Novelli G., Tonellini G., Mazzoleni F., Bozzetti A., Sozzi D. (2014). Virtual surgery simulation in orbital wall reconstruction: Integration of surgical navigation and stereolithographic models. J. Craniomaxillofac. Surg..

[40] Novelli G., Gramegna M., Tonellini G., Valente G., Boni P., Bozzetti A., Sozzi D. (2016). Orbital Osteoblastoma: Technical Innovations in Resection and Reconstruction Using Virtual Surgery Simulation. Craniomaxillofac. Trauma Reconstr..

[41] Bell R.B., Weimer K.A., Dierks E.J., Buehler M., Lubek J.E. (2011). Computer planning and intraoperative navigation for palatomaxillary and mandibular reconstruction with fibular free flaps. J. Oral Maxillofac. Surg..

[42] Shan X.F., Chen H.M., Liang J., Huang J.W., Zhang L., Cai Z.G., Guo C. (2016). Surgical navigation-assisted mandibular reconstruction with fibula flaps. Int. J. Oral Maxillofac. Surg..

[43] Heiland M., Habermann C.R., Schmelzle R. (2004). Indications and limitations of intraoperative navigation in maxillofacial surgery. J. Oral Maxillofac. Surg..

[44] Casap N., Wexler A., Eliashar R. (2008). Computerized navigation for surgery of the lower jaw: Comparison of 2 navigation systems. J. Oral Maxillofac. Surg..

[45] Widmann G., Keiler M., Zangerl A., Stoffner R., Longato S., Bale R., Puelacher W. (2010). Computer-assisted surgery in the edentulous jaw based on 3 fixed intraoral reference points. J. Oral Maxillofac. Surg..

